# Origin of the Chemical and Kinetic Stability of Graphene Oxide

**DOI:** 10.1038/srep02484

**Published:** 2013-08-21

**Authors:** Si Zhou, Angelo Bongiorno

**Affiliations:** 1 School of Chemistry and Biochemistry, Georgia Institute of Technology, 901 Atlantic Drive, Atlanta, Georgia, 30332-0400, USA; 2 School of Physics, Georgia Institute of Technology, Atlanta 30332-0430, Georgia, USA

## Abstract

At moderate temperatures (≤ 70°C), thermal reduction of graphene oxide is inefficient and after its synthesis the material enters in a metastable state. Here, *first-principles* and statistical calculations are used to investigate both the low-temperature processes leading to decomposition of graphene oxide and the role of ageing on the structure and stability of this material. Our study shows that the key factor underlying the stability of graphene oxide is the tendency of the oxygen functionalities to agglomerate and form highly oxidized domains surrounded by areas of pristine graphene. Within the agglomerates of functional groups, the primary decomposition reactions are hindered by both geometrical and energetic factors. The number of reacting sites is reduced by the occurrence of local order in the oxidized domains, and due to the close packing of the oxygen functionalities, the decomposition reactions become – on average – endothermic by more than 0.6 eV.

Graphene oxide (GO)^1^,^2^ is a material with potential applications in ultra-thin electronics^3^,^4^, optoelectronics^5^,^6^, energy storage^7^,^8^,^9^,^10^, sensor devices^11^, and mechanical actuators^12^,^13^. GO is generally obtained through the use of strong acid/base treatments^4^,^5^,^6^,^7^,^8^,^9^,^10^,^11^,^12^,^13^,^14^,^15^,^16^, resulting in the addition of oxygen in the forms of epoxide and hydroxyl species to the un-defected regions of the carbon layers, and carboxyl, carbonyl, and phenol groups to the edges of the carbon sheets^17^. As-synthesized GO is nonstoichiometric and hygroscopic, typically presenting O:C ratios between 0.3 and 0.5, and an uncertain but variable amount of water molecules intercalated between the oxidized carbon layers^18^,^19^,^20^. Synthesized for the first time 150 years ago^21^, GO remains still today an elusive material system whose properties are difficult to control.

Controlling the chemical structure of GO is crucial to, for instance, opening up a band gap for enabling applications in electronics^3^,^4^, or to calibrate its catalytic^11^,^22^ and mechanical properties^12^,^13^,^23^. Most of the efforts to control GO rely today on post-synthesis chemical and thermal treatments^24^,^25^,^26^,^27^,^28^,^29^,^30^,^31^, favoring the occurrence of reduction mechanisms, loss of oxygen content, and thus partial restoration of graphene-like properties. Thermal reduction of GO, in particular, initiates at temperatures larger than 70°C^30^, it leads to the desorption of H_2_O and O_2_, but also CO and CO_2_^30^,^32^, and consequently it is detrimental to the structural integrity of the graphene sheets and the physical properties of the reduced material. At temperatures lower than 70°C, reduction mechanisms become inefficient or die off, GO enters in a metastable state, and after its synthesis the material is driven towards a quasi-equilibrium state with a stable O:C ratio and structure^33^,^34^. While reduction mechanisms of GO and reduced GO have been investigated extensively in recent years^6^,^25^,^35^,^36^,^37^,^38^, the stability and structure of GO aged or annealed at moderated temperature (≤ 70°C) have received much less attention to date. Computations based on the density functional theory, in particular, have been used to generate and study model structures of GO^39^,^40^,^41^,^42^, to interpret experimental measurements^33^,^34^,^43^,^44^, and to elucidate reduction mechanisms^38^,^42^,^45^ and the thermodynamic stability of GO^46^,^47^. In spite of efforts, however, the origin of the chemical and kinetic stability of GO remains elusive to date.

Here, we perform density functional theory (DFT) calculations, we develop an alternative and simplified energy scheme describing GO, and then we combine both types of calculations to elucidate the mechanisms underlying the chemical and kinetic stability of GO. In this work, we consider the case of graphene layers functionalized with epoxide and hydroxyl groups, and thus we investigate the stability of large-area GO sheets^33^,^34^,^48^. This study shows that the key factor underlying the stability of GO is the tendency of the oxygen functionalities to diffuse, agglomerate, and form highly oxidized domains surrounded by regions of pristine graphene. Within these agglomerates, spontaneous decomposition reactions producing O_2_ or H_2_O molecules are hindered by both geometrical and energetic factors. In detail, the number of sites where pairs of oxygen functional groups can meet and react is reduced by the occurrence of local order in the highly oxidized domains, and due to the close packing of oxygen functional groups, at these sites the decomposition reactions become endothermic by – on average – 0.6 eV. Overall, this study shows that both O:C ratio and ageing processes can be used to enhance the chemical stability of GO, and therefore that aging as-synthesized GO at moderate temperatures may consist of a simple post-synthesis treatment to temper and control material properties, or produce new forms of GO.

## Results

**Figure 1:**
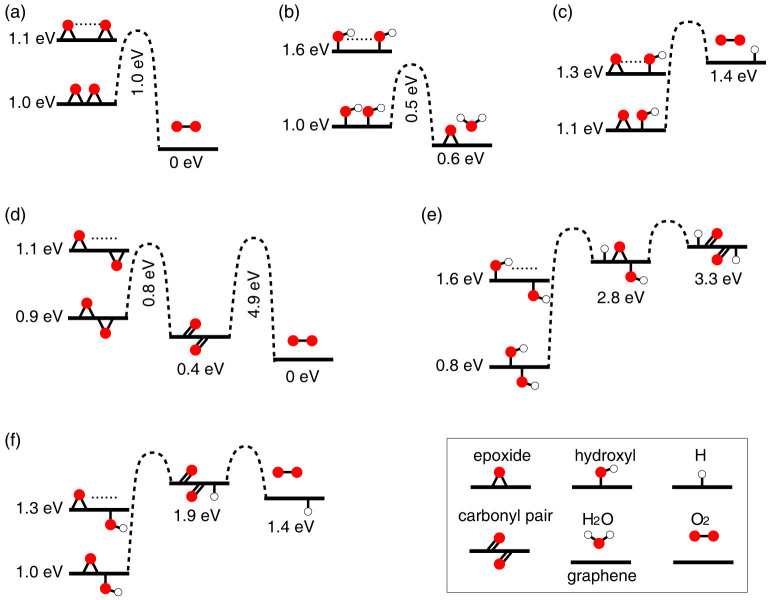
Binary decomposition reactions between epoxide and hydroxyl groups on graphene. (a) Epoxide-epoxide reaction leading to formation of O_2_. (b) Hydroxyl-hydroxyl reaction leading to formation of a H_2_O molecule and an epoxide group on graphene. (c) Hydroxyl-epoxide reaction leading to formation of O_2_ and a hydrogen species chemisorbed on graphene. The reactions in (a), (b), and (c) involve oxygen species chemisorbed on the same side of the basal plane. (d) Two epoxides chemisorbed on opposite sides of a graphene layer forming a carbonyl-pair and then O_2_. (e) Two hydroxyls on opposite sides of graphene transforming into a carbonyl-pair and hydrogen species. (f) An epoxide and a hydroxyl on opposite sides of the layer transforming into molecular oxygen and a hydrogen species. Energy values correspond to ΔG values *per* oxygen group computed via Eq. (2). Relevant energy barriers are computed by using NEB-DFT calculations. Bottom-right corner, legends of symbols used to represent reagents, by-products, and final products.

Figure 1 shows the possible elementary binary reactions between epoxide and hydroxyl species chemisorbed on a graphene layer leading to desorption of O_2_ or H_2_O molecules. The reaction energy diagrams shown in Fig. 1 are derived from DFT calculations^49^. Transition states are calculated by using nudged elastic band (NEB) DFT calculations^49^. Technical details of the calculations (see Methods) and the full list of results are reported and discussed in the Electronic Supplementary Information (ESI). Throughout this work, free energy values of GO are referred to that ones of pristine graphene and a gas of O_2_ and H_2_O molecules at room temperature and standard pressure. In detail, the chemical potentials of O_2_ and H_2_O are set equal to: 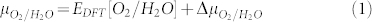
where E_DFT_ is the zero-temperature molecular energy computed from DFT, and Δμ is an experimental correction accounting for the entropic and enthalpic contributions to the chemical potential of these gaseous molecular species^50^,^51^. The Gibbs free energy of formation, ΔG, of an arbitrary distribution of N_e_ epoxides and N_h_ hydroxyl groups on graphene is defined as: 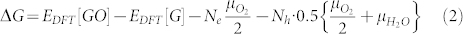
where E_DFT_[GO] and E_DFT_[G] are the total energies computed by using our DFT scheme of defected and pristine graphene layers, respectively. Enthalpic and entropic contributions to ΔG associated to defect formation were estimated by using standard methods^51^ in the case of a single epoxide species on graphene, a single hydroxyl species on graphene, and selected GO models consisting of disordered distributions of epoxide and hydroxyl species on graphene. These calculations show that, at room temperature and 1 atm, enthalpic and (vibrational) entropic corrections to ΔG in Eq. (2) amount to less than −0.1 eV *per* oxygen defect. For completeness, this latter energy value multiplied by the total number of oxygen functionalities is added to the right side of Eq. (2), thereby leading to an approximate but appropriate definition of ΔG. In Fig. 1 and throughout this work, values of ΔG are given *per* oxygen functional group. We note that the Gibbs free energies of formation of a single epoxide or hydroxyl group on graphene are found to be equal to about 1.1 eV and 1.6 eV, respectively (Fig. 1). These energy values indicate that sparse and homogeneous functionalizations of graphene with epoxide and hydroxyl groups are chemically unstable and prone to decompose *via* one of the binary reactions shown in Fig. 1 into O_2_, H_2_O, and pristine graphene.

The free-energy diagrams in Fig. 1 show that the most efficient – in energetic terms – low-temperature decomposition reactions of GO producing O_2_ and H_2_O involve either two epoxides or two hydroxyls chemisorbed on the same side of the carbon basal plane. The reaction between the two epoxides is exothermic by more than 1 eV, it is controlled by an energy barrier of about 1 eV, and it involves an electronic singlet-to-triplet spin transition (ESI), while that one between the two hydroxyls is exothermic by about 0.5 eV and it involves energy barriers of about 0.5 eV (Fig. 1). The remaining binary reactions between epoxides and hydroxyl groups lead to the formation of stable intermediate species that, with the exception of the carbonyl-pair group formed by the reaction of two epoxides chemisorbed on opposite sides of a graphene layer, lie higher in energy than the reagents by more than 1 eV. Carbonyl-pair groups are energetically more stable than two individual epoxide species on graphene (Fig. 1), and their transformation into O_2_ involves energy barriers as high as 4.9 eV, comparable to the energy costs required by oxygen functionalities to break through the graphene network and switch the side onto which they are chemisorbed. Thus, at moderate temperatures these reactions are unlikely to constitute viable pathways leading to the decomposition of GO, which therefore is dominated by the reactions in Figs. 1(a) and 1(b).

In addition to kinetic information, Figure 1 reports the energy gains yielded by associating two individual oxygen functionalities on graphene into a dimeric species formed by epoxide and/or hydroxyl groups sharing a C = C bond. The energy gains show, in particular, that the interaction between epoxide and hydroxyl species on graphene is, in all cases, attractive. The attraction is largest for hydroxyls, while for all possible combinations the association energy is larger when the two oxygen groups are chemisorbed on the opposite rather than the same side of the carbon basal plane. The kinetics of agglomeration is governed by diffusion processes, and in case of isolated epoxide and hydroxyl groups on graphene, their diffusion rates are controlled by migration energies of 0.8 eV and 0.5 eV, respectively (ESI). A number of DFT studies have addressed the diffusion and interaction of epoxide and hydroxyl species on graphene, and the results thus far discussed are in agreement with these recent computational works^25^,^37^,^39^,^40^,^42^,^47^,^52^,^53^,^54^,^55^,^56^. Here, we address in detail the effects played by agglomeration phenomena onto the chemical stability and structure of GO.

**Figure 2:**
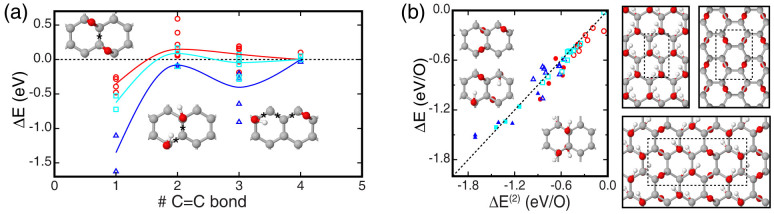
Pair-wise association energy of oxygen functional groups on graphene. (a) Energy computed using DFT (symbols) of binary epoxide-epoxide (red), hydroxyl-hydroxyl (blue), and epoxide-hydroxyl (cyan) complexes with functional groups separated by up to 4 C = C bonds. Energy values are obtained by referring the energy of the binary complex to that ones of pristine graphene and the isolated species on graphene. Insets show binary complexes having functional groups separated by one, two, and three C = C bonds (asterisks). Various non-equivalent binary complexes can be formed with oxygen functionalities separated by a selected number of C = C bonds (ESI). Solid colored lines are guide to the eye. (b) Energy of single-layer GO models presenting epoxy-only (red), hydroxyl-only (blue), and mixed epoxide-hydroxyl (cyan) functionalizations as computed from both DFT and the simplified energy scheme in Eq. (3). Values of ΔE (*per* functional group) computed using DFT are obtained by referring the energy of GO to that ones of pristine graphene and the isolated species on graphene. Filled symbols refer to crystalline structures with high O:C ratios, while empty symbols show the energy of sparse agglomerates of functional groups on graphene with low O:C ratios. Insets and ball and stick illustrations on the right side show selected GO structures considered in these comparisons; C, O, and H are shown in gray, red, and white colors (the full list of model structures is reported in the ESI).

Figure 2(a) shows the association energy of pairs of nearest neighbor epoxides, hydroxyls, and epoxide and hydroxyl groups chemisorbed on graphene. We consider, in particular, non-equivalent complexes formed by two oxygen functional groups, epoxide or hydroxyl species, separated by up to four C = C bonds and chemisorbed on either the same or opposite side of a basal plane. Our DFT calculations show that, in all cases, the two-species interaction is – on average – attractive and short-range, decaying to negligible values when the two oxygen groups are separated by more than 4 C = C bonds (Fig. 2(a)). The association energies of pairs of oxygen functional groups separated by less than 4 C = C bonds are scattered over wide energy intervals (> 0.5 eV), indicating that the binding energy of such binary complexes results from a non-trivial competition of strain and electronic effects. The two-species complexes and corresponding binding energies in Fig. 2(a) are here used to devise a simplistic and approximated energy scheme, alternative to DFT, and suited to describe arbitrary epoxide/hydroxyl functionalizations of graphene.

We express the energy of a GO layer as sum of two-species energy contributions E_IJ,K_, where IJ refers to a pair of oxygen functional groups separated by less than 4 C = C bonds, and K refers to its corresponding binary association energy in Fig. 2(a). In detail, we write: 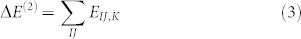
where ΔE^(2)^ is the total energy of the GO layer relative to the energy of individual oxygen functional groups on graphene. Based on Eq. (3), we can thus define the Gibbs free energy of formation of a GO layer hosting N_e_ epoxides and N_h_ hydroxyls as: 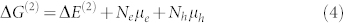
where μ_e_ and μ_h_ are the Gibbs free energy of formation computed using DFT of a single epoxide and hydroxyl species on graphene, respectively. To test and calibrate this simple energy scheme, we use reference energies computed using DFT of an extended set of single-layer GO models, including model structures consisting of small agglomerates of a few epoxide or hydroxyl species on graphene to ordered and homogeneous structures of a GO layer presenting varying O:C ratios (ESI). The comparison between the energies obtained by using DFT and the simplified additive scheme shows that Eq. (3) reaches a good accuracy provided that, one, we exclude from the set of binary association energies reported in Fig. 2(a) that ones corresponding to epoxide-epoxide pairs exhibiting mild repulsive interaction energies (< 0.3 eV), and two, we add a penalty energy of 0.3 eV to each energy term E_IJ,K_ associated to hydroxyl-hydroxyl complexes. With these two *ad hoc* corrections, our simplistic scheme reproduces energy values derived from DFT calculations of a variety of GO models with a mean absolute error of less than 0.1 eV (Fig. 2(b)). These comparisons also show that the aforementioned *ad hoc* corrections are necessary to account for – approximately and on average – the decreasing and increasing strain energy of agglomerates containing more than a pair of epoxide or hydroxyl groups, respectively (ESI). We use the aforementioned simplified energy scheme to predict the structure and energy of GO layers aged at mild temperatures (see Methods).

**Figure 3:**
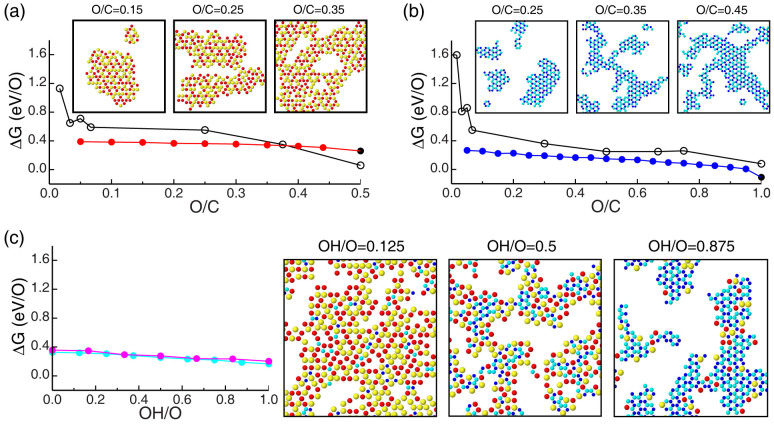
Chemical stability and structure of large-area GO sheets. (a) ΔG *per* functional group of GO layers oxidized with only epoxide species. Black symbols connected by segments show values of ΔG computed using DFT and *hand-made* model structures of GO, encompassing small clusters and regular distributions of epoxide species of varying O:C ratio. Red symbols connected by segments show values of ΔG of GO models generated from Monte Carlo simulated annealing simulations and thus mimicking aged structures of this material with varying O:C ratios. Insets show a few selected model structures of aged GO functionalized with only epoxide groups. In these illustrations, graphene is not shown and red and yellow discs indicate epoxide species facing upward and downward the basal plane, respectively. (b) Same as (a) for hydroxyl-only functionalizations of graphene. In the insets, hydroxyl species facing upward and downward the carbon plane are shown in blue and cyan, respectively. (c) ΔG *per* functional group of GO layers oxidized with both epoxide and hydroxyl group. OH/O refers to the fraction of hydroxyl groups relative to the total amount of oxygen. The GO models are generated via Monte Carlo simulations (see Methods). Magenta and cyan symbols (connected by segments) refer to GO models with fixed O:C ratios equal to 0.3 and 0.4, respectively. The illustrations on the right side show selected configurations of aged GO layers with an O:C ratio of 0.4 and different relative fractions of hydroxyl and epoxide species.

Figures 3(a) and 3(b) report values of ΔG *per* functional group of epoxy- and hydroxyl-only functionalizations of graphene, respectively. Figure 3(a) reports Gibbs free energy values derived from Eq. (2), DFT calculations, and GO models presenting regular and homogeneous distributions of oxygen functional groups (ESI), while Figure 3(c) reports energy values derived from Eq. (4), Monte Carlo simulated annealing simulations, and thus model structures of GO obtained by mimicking ageing at moderate temperatures (see Methods and ESI). Although greater than zero, the free energy values in Fig. 3(a) and 3(b) show that the chemical stability of GO in standard ambient conditions increases with the O:C ratio, nearly reaching equilibrium with the gas of O_2_ and H_2_O molecules in ambient conditions in the case of fully oxidized and crystalline structures of GO. Furthermore, our Monte Carlo simulations show that, regardless the type of oxygen functionalization, ageing leads to the agglomeration of functional groups, enhanced chemical stability, and formation of a non-homogeneous phase of GO consisting of highly oxidized domains surrounded by areas of pristine graphene (Fig. 3). Similar results are obtained in the case of mixed epoxide-hydroxyl functionalizations of graphene (Fig. 3(c)). In this case, in addition to aggregation phenomena, ageing favors also segregation phenomena and formation within the highly oxidized domains of regions rich in epoxide and hydroxyl groups (Fig. 3(c)). DFT and lattice model Monte Carlo simulations therefore show that, due to the attractive interaction between oxygen functional groups on graphene, both O:C ratio and ageing at mild temperature enhance chemical stability of GO and favor the appearance of partial structural order.

## Discussion

**Figure 4:**
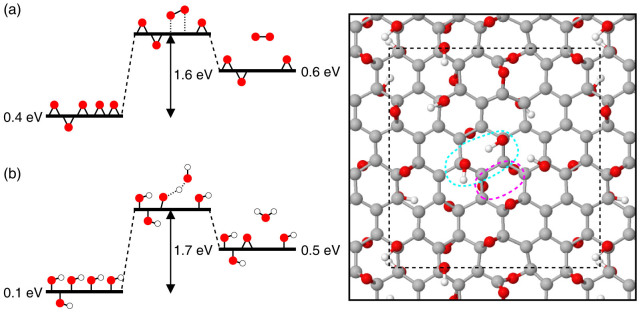
Binary decomposition reactions of aged GO. (a) Schematic energy diagram of the binary reaction between epoxide groups embedded in oxidized domains presenting high concentrations of epoxide groups. Values of ΔG (*per* functional group) of reagents and products correspond to averages extracted from the model structures of aged GO generated by Monte Carlo simulations and the approximated energy scheme in Eq. (3). The energy barrier of 1.6 eV shown in (a) refers to the energy value extracted from a NEB-DFT calculation using the model structure of GO on the right and the pair of reacting epoxide groups circled with the dashed magenta line. (b) Same as (a) in the case of two hydroxyls embedded in an oxidized region of aged GO layers rich in hydroxyl groups. Also in this case, values of ΔG for reagents and products are derived by using the energy scheme of Eq. (3) and model structures of aged GO. The energy barrier of 1.7 is extracted from a NEB-DFT calculation using the GO model shown on the right and the pair of reacting hydroxyl groups circled by the dashed cyan line. In the ball and stick illustration, the dashed box shows the planar dimensions of the supercell used in the DFT calculations. More details about this model structure and NEB-DFT calculations are reported in the ESI.

Our DFT and Monte Carlo calculations show that, in spite of the enhanced chemical stability, GO aged at moderate temperatures does not reach equilibrium with the gas phase and thus remains susceptible to decompose into gas molecules and pristine graphene (Fig. 3). Our calculations show, however, that aggregation phenomena and the formation of a non-homogeneous phase of oxidized and non-oxidized domains influence not only the chemical but also the kinetic stability of GO. In fact, the analysis of the models of aged GO generated *via* Monte Carlo simulations shows that within the highly oxidized regions all the decomposition processes shown in Fig. 1 become – on average – endothermic, including the reactions between two nearest neighbor epoxides or hydroxyls chemisorbed on the same side of the basal plane; the energy diagrams of these two decomposition reactions are shown in Fig. 4. In these diagrams, the free energy of the initial state corresponds to ΔG of aged GO, that one of the final state is obtained by averaging the energy costs associated to decomposition reactions of eligible pairs of oxygen functionalities, while energy barriers are extrapolated from NEB-DFT calculations based on model structures of GO presenting a disordered distribution of epoxide and hydroxyl species (ESI). Our analyses – of a variety of model structures of aged GO generated by Monte Carlo simulated annealing simulations – show that within the highly oxidized regions, local order and spatial correlations reduce the fractions of epoxide and hydroxyl groups eligible to react to approximately 0.3 and 0.2, respectively. The average heats of reaction of pairs of epoxides and hydroxyls forming O_2_ and H_2_O are about 0.6 eV and 0.8 eV, respectively. Further, our NEB-DFT calculations show that the energy barriers associated to the reactions shown in Fig. 4 attain, in the interior of oxidized domains, values larger than 1.6 eV. These results concur in showing that the origin of the kinetic stability of graphene oxide stems from the attractive interaction between the oxygen functional groups. These interactions stabilize energetically the pairs of reacting species susceptible to form O_2_ or H_2_O, rendering the decomposition processes endothermic and thus unlikely.

In summary, our *first-principles* calculations show that the most effective low-temperature decomposition reactions of GO are between pairs of epoxides or hydroxyls chemisorbed on the same side of graphene, that the interaction between oxygen functionalities on graphene is short-range and attractive, and that a simple and qualitatively correct energy scheme can be used to describe epoxy and/or hydroxyl functionalizations of graphene. *First-principles* and lattice model Monte Carlo simulations show that the key factor underlying the stability of GO is the tendency of the oxygen functionalities to diffuse, agglomerate, and form highly oxidized domains surrounded by regions of pristine graphene. Within these agglomerates, all the binary decomposition reactions producing O_2_ or H_2_O molecules are hindered by both geometrical and energetic factors. In detail, the number of sites where pairs of oxygen functional groups can meet and react is reduced by the occurrence of local order in the highly oxidized domains, and due to the close packing of oxygen functional groups, at these sites any decomposition reaction becomes endothermic by – on average – more than 0.6 eV. Overall, this study shows that both O:C ratio and ageing processes can be used to enhance both chemical and kinetic stability of GO. Aging as-synthesized GO at moderate temperatures may therefore consist of a simple post-synthesis treatment to both temper and control material properties, as well as to lead to new forms of GO.

## Methods

Density functional theory calculations are performed by using the QUANTUM-Espresso software^49^. We use an energy cutoff of 70 Ry, norm-conserving pseudopotentials^57^ for all atomic species, and the parametrization of the exchange and correlation energy functional proposed by Perdew, Burke, and Ernzerhof^58^. Test calculations show that cutoff energy, k-point mesh in the Brillouin zone, and size and geometry of supercells introduce errors of about ± 0.1 eV on our energy values. Bond energies of O_2_, CO, and H_2_O given by this *first principles* scheme are 5.3 eV, 10.9 eV, and 4.8 eV, respectively, within 3% of experimental data. *First principles* calculations of crystalline structures of GO are based on the use of small unit cells and dense k-point meshes, while the rest of the calculations are carried out by using supercells with planar dimension equal or larger than 5 × 6 graphene unit cells and by sampling the Brilloing zone in the Γ-point. The vacuum region separating the periodic graphene layers is – in all cases – equal to 12 Å.

Binding energy values and geometries of the binary complexes used to calculate *via* Eq. (3) the energy of an arbitrary distribution of epoxide and hydroxyl species chemisorbed on graphene are reported in the ESI. This simplified energy scheme is used in conjunction with lattice model representations of functionalized graphene and kinetic Monte Carlo simulations. In this numerical framework, epoxide- and hydroxyl-like defects occupy C = C bonds and C sites on one of the two opposite sides of the graphene plane, and Eq. (3) is used to compute the energy of a distribution of oxygen defects. The functional groups can move freely – with no energy barriers – between nearest neighbor defect sites. A Monte Carlo step consists in displacing a functional group selected at random to an empty nearest neighbor defect site, this one also selected at random. The energies computed by using Eq. (3) of the initial and modified defect distributions are used to calculate a Boltzmann factor, and thus accept or reject the new configuration. This lattice model Monte Carlo scheme is used in conjunction with simulated annealing simulations to mimic the ageing of GO and predict the structure resulting from the agglomeration of homogeneous distributions of functional groups (ESI). These Monte Carlo simulations are carried out by using large models of GO, including hundreds of oxygen functionalities. Number and nature of the oxygen functional groups are held fixed in the simulations.

It is to be noted that in our Monte Carlo simulations, temperature is a numerical parameter used to control (accelerate and/or arrest) the occurrence of structural transformations in our GO models. Temperature in our Monte Carlo simulations is thus not intended to mimic any experimental temperature. Due to the nature of the underlying energy scheme – which accounts only for the interaction of hydroxyl and epoxide species on a graphene layer, raising the temperature within our conventional Monte Carlo scheme does not introduce any new physics or chemistry besides that one described by the discrete energy scheme, and the simulated annealing simulations correspond simply to a numerical strategy used to speed up structural transformations and determine – by a slow quenching – a low free-energy arrested state of GO. Therefore, our Monte Carlo simulations have no direct link with the experiment; they are simply used to speed up the evolution (ageing) of GO in a regime where structural transformations are driven only by the diffusion of the epoxides and hydroxyls, i.e. moderate (experimental) temperatures.

## Author Contributions

S.Z. carried out calculations and analyses. A.B. supervised the research work and wrote the manuscript. Both authors reviewed the manuscript.

## Supplementary Material

Supplementary Information Supplementary Information: Origin of the Chemical and Kinetic Stability of Graphene Oxide
