# Supplementary material for: Origin of the Chemical and Kinetic Stability of Graphene Oxide
Source: Sci Rep. 2013 Aug 21;3:2484. doi: 10.1038/srep02484 (PMC3748429; doi:10.1038/srep02484)
Supplement: Supplementary Information — Origin of the Chemical and Kinetic Stability of Graphene Oxide [file srep02484-s1.pdf]

Supplementary Information for: Origin of the Chemical and Kinetic Stability of Graphene Oxide

Si Zhou[1,2] and Angelo Bongiorno*[1]

July 31, 2013

[1]*School of Chemistry & Biochemistry, Georgia Institute of Technology, Atlanta, Georgia 30332-0400*
[2]*School of Physics, Georgia Institute of Technology, Atlanta, Georgia 30332-0430*

In this work, we used the QUANTUM-Espresso toolkit [1] to perform our density functional theory (DFT) calculations. We used a plane-wave energy cutoff of 70 Ry, norm-conserving pseudopotentials [2] for all atomic species, and the exchange and correlation energy functional proposed by Perdew, Burke, and Ernzerhof [3]. We employed supercells of various dimensions. Depending on system size, we performed calculations based on either dense k-point meshes or only the Γ-point to sample the Brillouin zone of the periodic system. We used both the CP and PWscf codes, depending on the calculation to be performed. The CP code was used to optimize models presenting structural complexity and/or disorder, while the PWscf code was used to carry out nudged elastic band (NEB) calculations [4]. Both codes were used to perform full optimizations (electronic states, ionic positions, and cell parameters) calculations. Extensive tests were performed, showing that technical details of our calculations and the technical differences in the algorithms used by CP and PWscf codes introduce errors in the energy values of less than 0.1 eV.

Lattice models and Monte Carlo simulations are described in the main test. In this type of simulations, the energy of a distribution of epoxide and hydroxyl species on graphene is computed by using the following simple formula:

$$E^{(2)} = \sum_{IJ} E_{IJ,K}, \tag{1}$$

where IJ refers to a pair of oxygen functional groups separated by less than 4 C=C bonds, K indicates one of the possible non-equivalent configurations belonging to this family, and $E_{IJ,K}$ corresponds to the binding energy of such a binary complex – referred to the energies of graphene and the individual species on graphene. The binary configurations

and corresponding energy terms $E_{IJ,K}$ included in Eq. (1) are shown in Figures S1, S2, and S3. Only the energies (and relative configurations) indicated by black segments are used in Eq. (1). It is to be noted that, the energies of stable hydroxyl-hydroxyl pairs reported in Figure S2 are all included in Eq. (1); each one of these terms, however, is corrected by adding a penalty energy term equal to 0.3 eV. As shown in Figure S4, this correction accounts for the fact that the energy of hydroxyl-hydroxyl pairs depends on the relative orientation of the two OH bonds. A penalty energy of 0.3 eV per hydroxyl-hydroxyl pair has been shown to account well for this conformational and energy variability in aggregates including more than two hydroxyl species. Overall, the simple corrected energy scheme of Eq. (1) reproduces results computed using DFT with an average error of 0.1 eV (see Figures S5, S6, and S7).

Figure S8 shows energy profiles of two nearest neighbor epoxide species reacting and forming an O_2 molecule. The two energy profiles were obtained from spin-polarized NEB-DFT calculations by constraining the total spin to be equal to zero and one. The crossing point between the singlet and triplet potential energy surfaces is also shown in Figure S8. We used these reaction energy profiles to estimate the probability, p_{s-t}, for the singlet-to-triplet spin conversion at the crossing point shown in Figure S8. In particular, we relied on the Landau-Zener theory [5] and used the following equation:

$$p_{s-t} = 2\left[1 - \exp\left(-\frac{V^2}{hv|F_s - F_t|}\right)\right], \quad (2)$$

where V is the spin-orbit matrix element between the triplet and singlet states of free O_2, v is the velocity of the O_2 center of mass at the transition state, and F_s and F_t are the forces at the transition state acting on a O_2 molecule in the singlet and triplet state, respectively. As in Ref. [6], we employed $V = 122$ cm^{-1} and v equal to root mean square velocity of a gaseous O_2 molecule at T=300K. F_s and F_t were derived from the energy curves shown in Fig. S8 by taking the gradient with respect to the distance between the graphene layer and the O_2 center of mass. With the above values for V, v, and F_s and F_t, Eq. 2 gives a probability value of $p_{s-t} = 0.0042$, indicating a poor rate of spin conversion per single reaction process.

Figure S9 shows the energy profiles obtained from NEB-DFT calculations of single epoxide and hydroxyl groups migrating between nearest neighbor stable configurations on graphene. These calculations show that the activation energies associated to the diffusion process of epoxide and hydroxyl groups is about 0.8 eV and 0.3 eV, respectively.

Figures S10 and S11 provide additional details about our Monte Carlo simulations and model structures of aged GO, while Figures S12 shows the model structures of GO and reacting pairs used to estimate the reaction energy barriers associated to O_2 and H_2O formation when the reacting species are surrounded by other functional groups. Figure S13 show the energy diagrams derived from these NEB-DFT calculations.

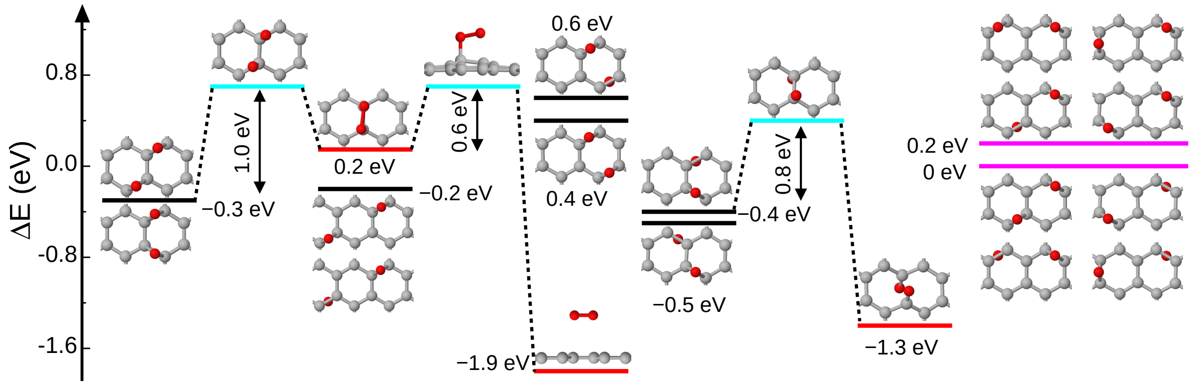

Figure **S1**: **Pair-wise association energy of epoxy groups on graphene**. Line segments show the energy of epoxide-epoxide complexes chemisorbed on a carbon basal plane; molecular configurations of epoxide-epoxide complexes are illustrated in the ball-and-stick images either above or below the line segments. C and O atoms are shown in gray and red colors, respectively. Energy values are referred to that ones of pristine graphene and isolated species on graphene. Cyan colored line segments indicate the transition states leading to the formation of either a O_2 molecule (left) or a carbonyl-pair species (right). The energy values shown by black line segments are employed as two-species energy contributions $E_{IJ,K}$ in our simplified additive scheme. Magenta line segments indicate the repulsive interaction energies excluded from the set of $E_{IJ,K}$ terms used in our simplified additive scheme.

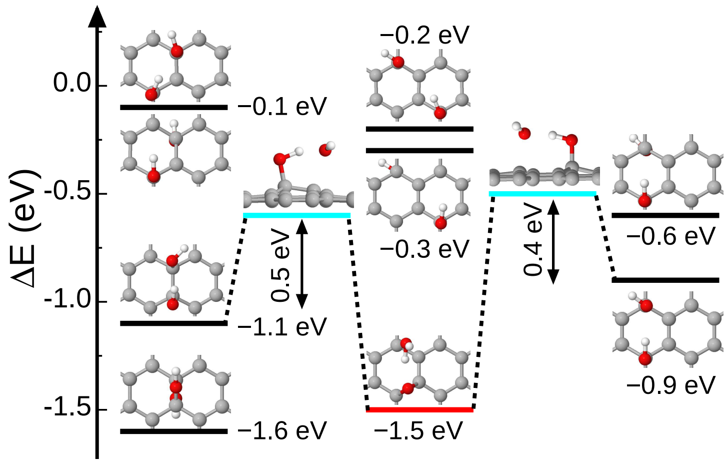

Figure **S2**: **Pair-wise association energy of hydroxyl groups on graphene**. Same as Figure S1 for hydroxyl-hydroxyl complexes on graphene. Cyan colored line segments indicate the transition states for two reacting hydroxyl groups in ortho-position (left) and para-position (right) leading to the formation of a H_2O molecule and an epoxy group. Energy values shown by black line segments and corresponding hydroxyl-hydroxyl pairs are included in our simplified additive scheme.

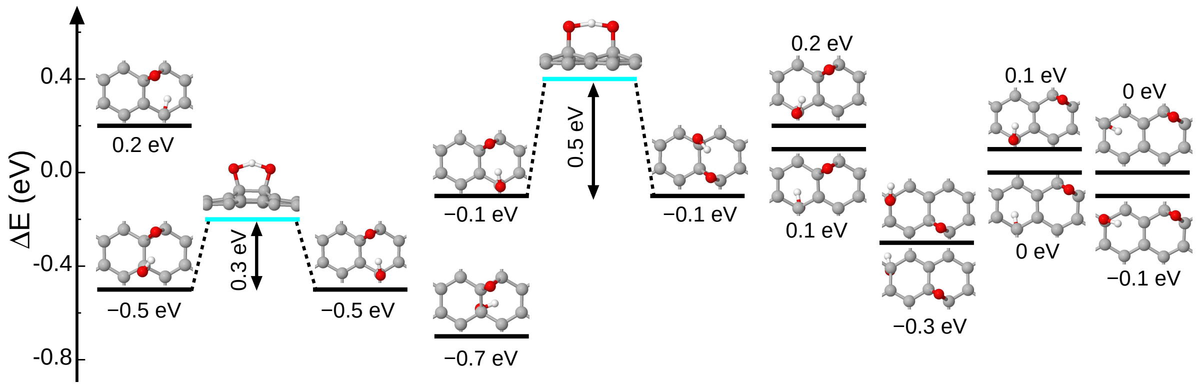

Figure **S3**: **Pair-wise association energy of an epoxide and a hydroxyl group on graphene.** Same as Figures S1 and S2 in the case of binary complexes formed by a epoxide and a hydroxyl group. Cyan colored line segments indicate the transition states leading to proton transfer between nearest neighbors hydroxyl and epoxide groups. Energy values shown by black line segments and corresponding hydroxyl-epoxide pairs are included in our simplified additive scheme.

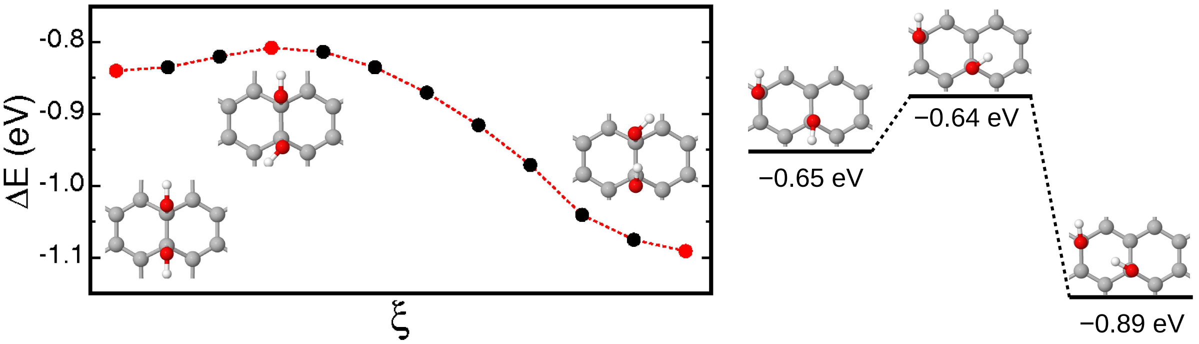

Figure **S4**: **Role of OH-bond orientation on the association energy of two nearest neighbor hydroxyl species**. Left panel, energy profile obtained from NEB-DFT calculations (symbols connected by dashed line) of two nearest neighbor hydroxyl species sharing a C=C bond whose OH-bonds undergo the rotations showed by the ball-and-stick images. Energies of the two stable configurations, shown on the left and right sides, and of the transition state, middle image, are indicated with red symbols. Right panel, stable configurations of two nearest neighbor hydroxyl species showing different relative orientation of the two OH bonds. Energy values are obtained from DFT calculations and are referred to that ones of pristine graphene and isolated species on graphene.

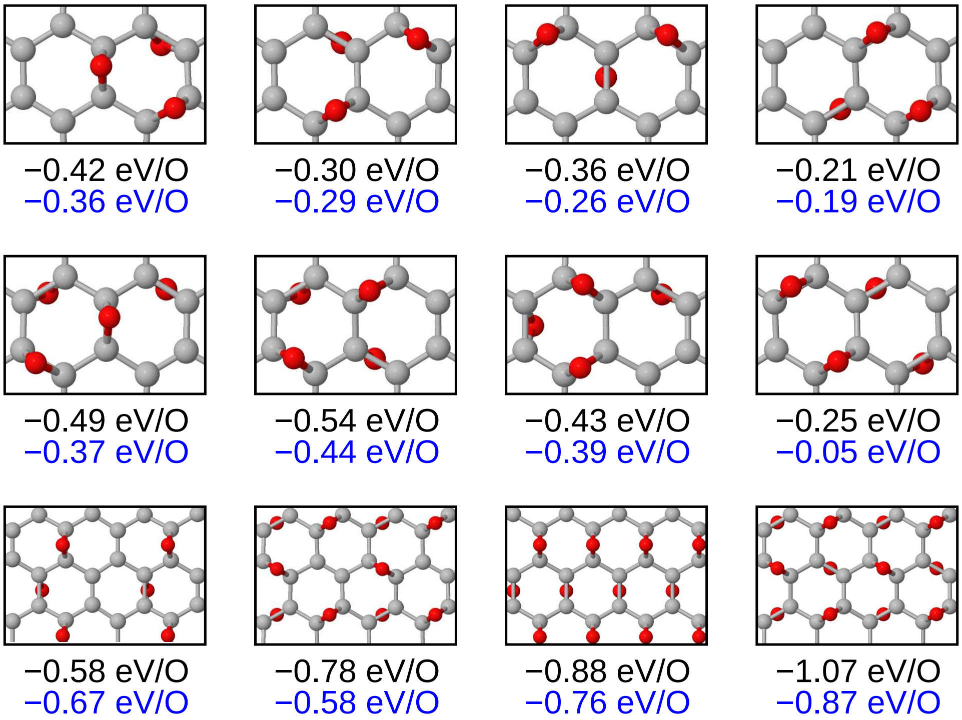

Figure **S5**: **DFT vs. additive energy scheme of Eq. (1): epoxy functionalizations of graphene**. Energy of trimer (top panels) and tetramer (middle panels) clusters, and of regular and homogeneous arrangements (bottom panels) of epoxide species on graphene as computed using DFT (black) and the energy scheme in Eq. (1) (blue). The O:C ratio of these model structures of GO is 0.05 (trimers), 0.067 (tetramers), and – bottom panels, from left to right – 0.25, 0.375, 0.5 and 0.5. Energy values (given per oxygen species) are referred to that ones of pristine graphene and a single epoxide on graphene.

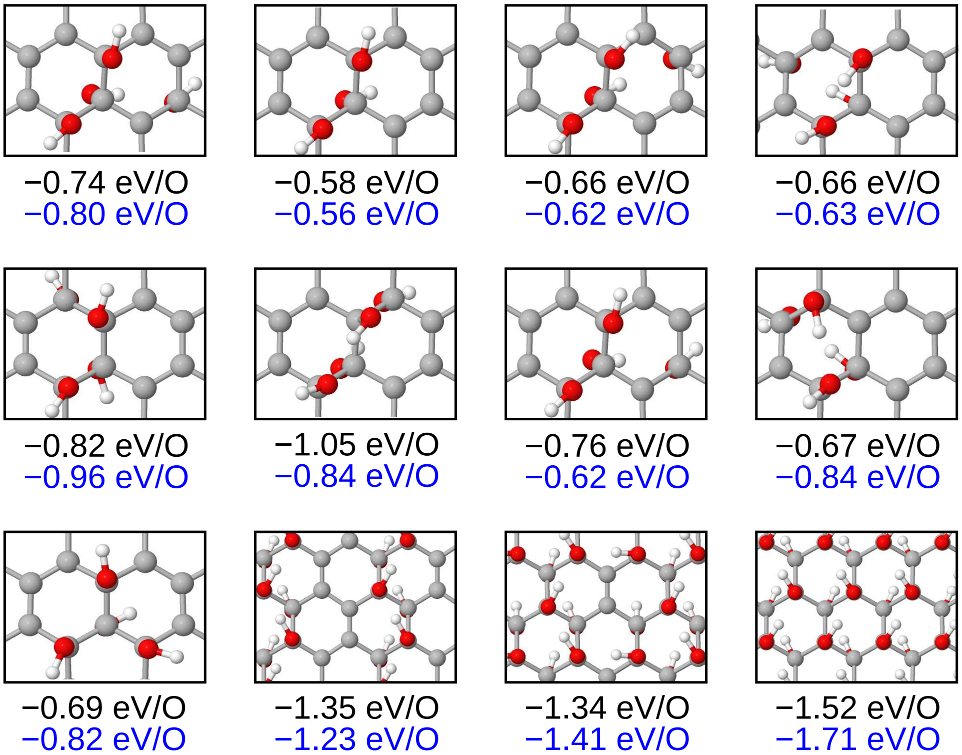

Figure **S6**: **DFT vs. additive energy scheme of Eq. (1): hydroxyl functionalizations of graphene**. Energy of trimer (the two leftmost panels on top), tetramer (the two rightmost panels on top, middle panels, and the leftmost panel on the bottom) clusters, and of regular and homogeneous arrangements (the three rightmost panels on the bottom) of hydroxyl species on graphene as computed using DFT (black) and the energy scheme in Eq. (1) (blue). The O:C ratio of these model structures of GO is 0.05 (trimers), 0.067 (tetramers), and – bottom panels, from left to right – 0.5, 0.75, and 1.0. Energy values (given per oxygen species) are referred to that ones of pristine graphene and a single hydroxyl on graphene.

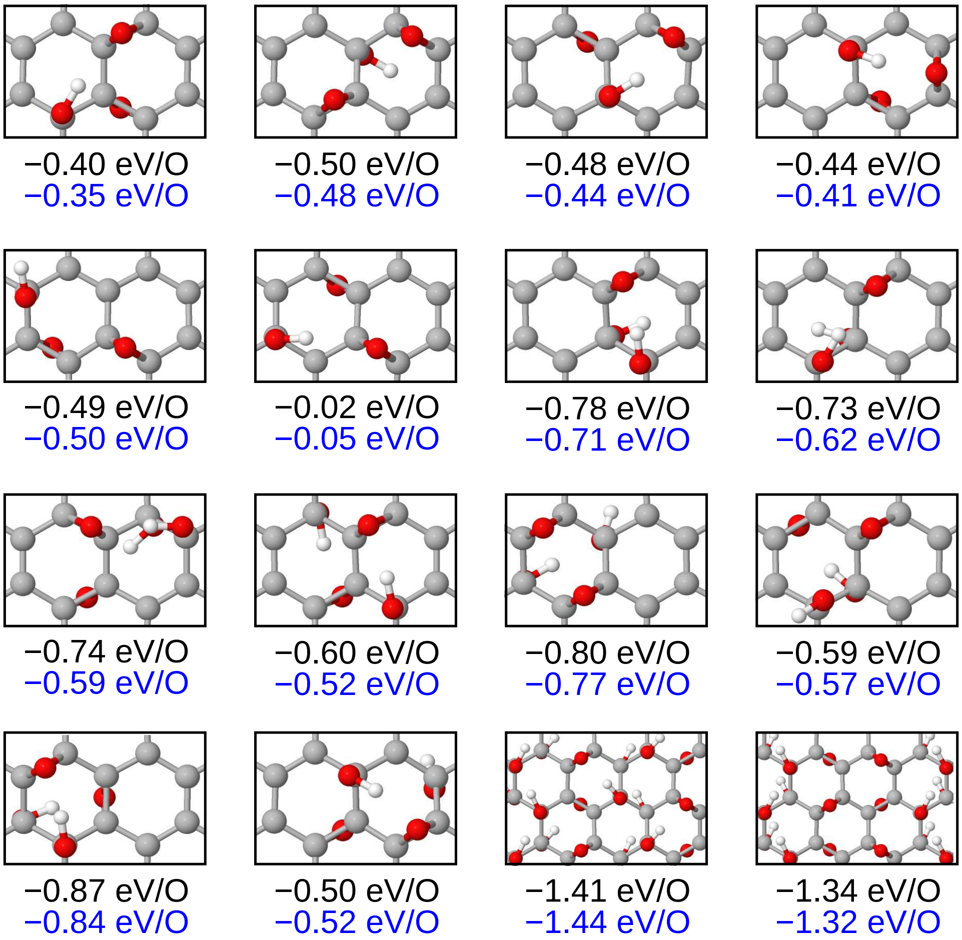

Figure **S7**: **DFT vs. additive energy scheme of Eq. (1): mixed epoxide-hydroxyl functionalizations of graphene**. Energy of trimer (the two topmost panels), tetramer (third row of panels from the top and the two leftmost panel on the bottom) clusters, and of regular and homogeneous arrangements (the two rightmost panels on the bottom) of epoxide and hydroxyl species on graphene as computed using DFT (black) and the energy scheme in Eq. (1) (blue). The O:C ratio of these model structures of GO is 0.05 (trimers), 0.067 (tetramers), and – bottom panels, from left to right – 0.75 and 0.67. In these two regular and homogeneous structures, the relative fraction of hydroxyl and epoxide groups is 2:1 and 1:1, respectively.

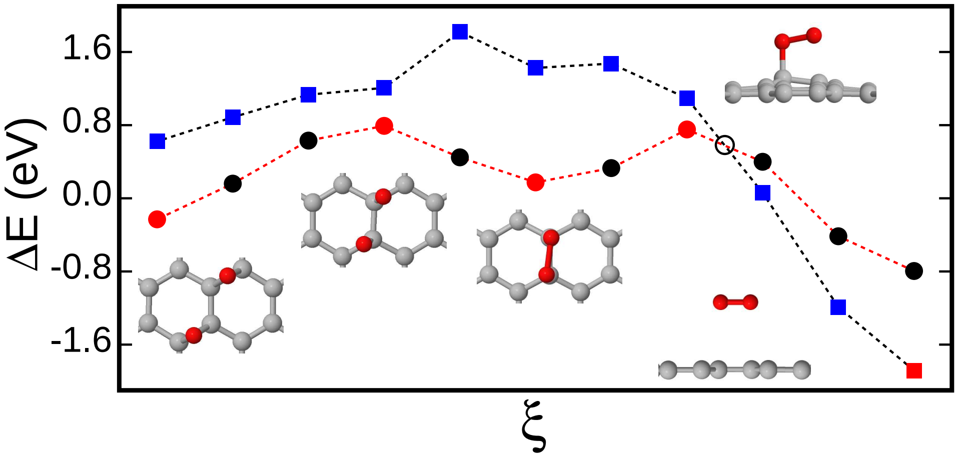

Figure **S8**: **Reaction between two nearest neighbor epoxide groups forming an O_2 molecule**. The reaction energy profiles are computed by spin-polarized NEB-DFT calculations (symbols connected by dashed lines) with the total spin moment constrained to be equal to zero (discs) and one (squares). Insets show stable and transition state configurations of the two-oxygen species reacting on graphene. The energies of these configurations are indicated by the red symbols nearby. Energy values are referred to that ones of pristine graphene and isolated species on graphene.

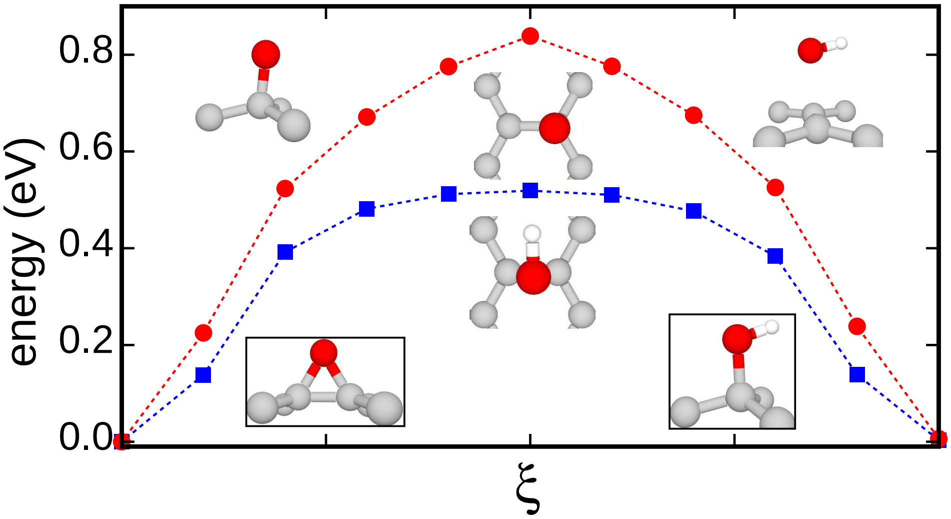

Figure **S9**: **Diffusion energy barriers of epoxide and hydroxyl groups on graphene**. Red (blue) symbols connected by dotted segments show the energy profile computed by NEB-DFT of an epoxide (hydroxyl) species migrating between two nearest neighbor stable configurations, respectively, Insets show stable configurations (within frames) and transitions states (top and side views).

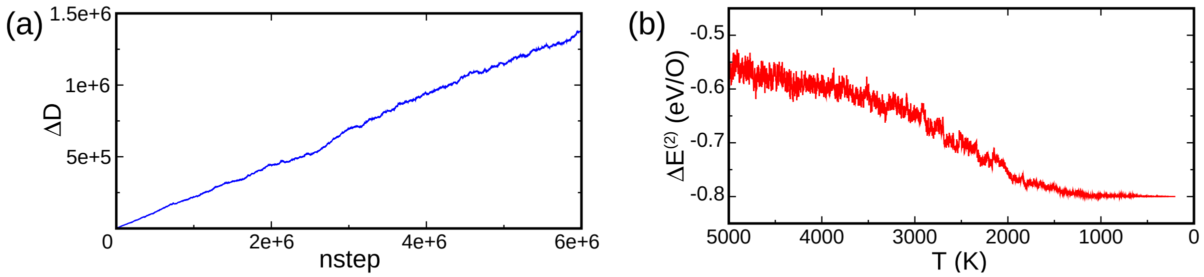

Figure **S10**: **Lattice model Monte Carlo simulated annealing simulations**. (a) Total square displacement vs. Monte Carlo steps of oxygen groups on a lattice model at a high temperature. Randomization of oxygen species on graphene at 5000 K was performed – in each case – by carrying out long Monte Carlo runs. This simulation was then followed by another long Monte Carlo simulation where the temperature was slowly quenched to 200 K. (b) Energy of a model GO computed by using Eq. (1) vs. T extracted from a Monte Carlo quenching simulation.

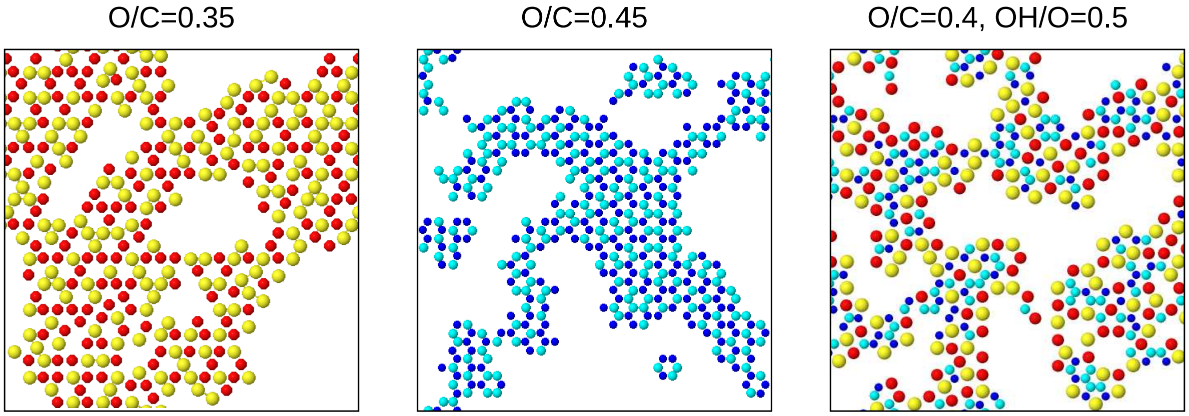

Figure **S11**: **Model structures of aged GO**. Model structures of aged GO presenting epoxy-only (left), hydroxyl-only (middle), and mixed epoxide-hydroxyl (right) functionalizations. The O:C ratio of the three models is, from left to right, 0.35, 0.45, and 0.4, respectively. The GO model presenting a mixed functionalization include equal fractions of epoxide and hydroxyl species. To mimic the ageing process, we used lattice model Monte Carlo simulated annealing simulations. Graphene is not shown and red and yellow discs indicate epoxide species facing upward and downward the basal plane, respectively. Blue and cyan discs indicate hydroxyl species facing upward and downward the basal plane, respectively.

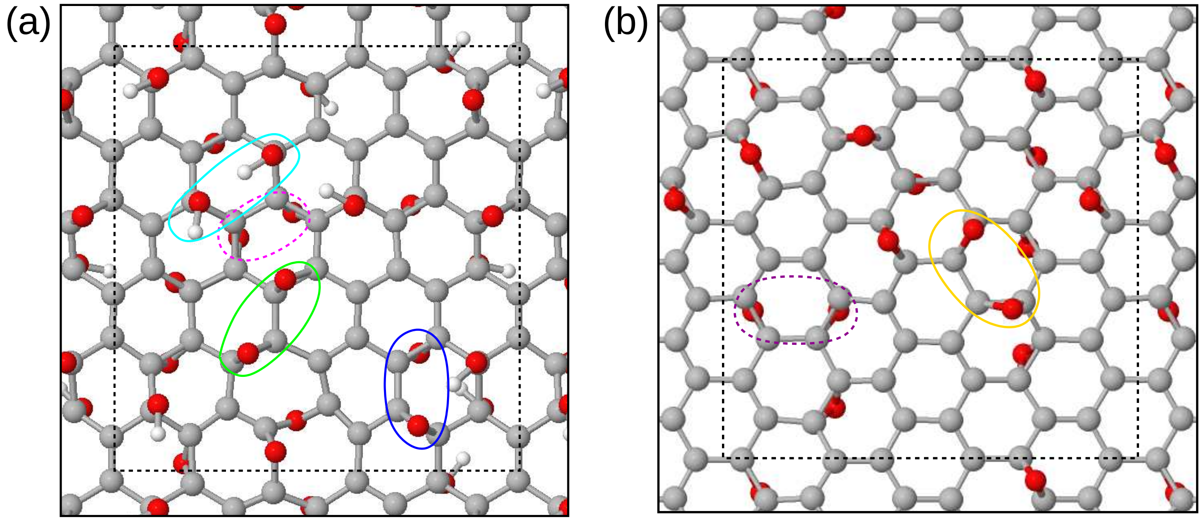

Figure **S12**: **Model structures of GO generated by DFT.** (a) GO model with an O/C ratio equal to 0.42 and a fraction of hydroxyls equal to 0.4. (b) GO model with an O/C ratio of 0.25 including only epoxide groups. Both models have been generated from DFT by using a periodic supercell with planar dimensions equal to 5×6 graphene unit cells (dashed frames) and, in the perpendicular direction, a vacuum region of 12 Å. The models include 60 C atoms, the O species were distributed at random, and DFT was used to optimize energy and ionic positions. Colored ellipses show pairs of oxygen species which can react to form O_2, H_2O (cyan), and a carbonyl-pair (blue). We used NEB-DFT calculations to compute the energy barrier of the six binary reactions.

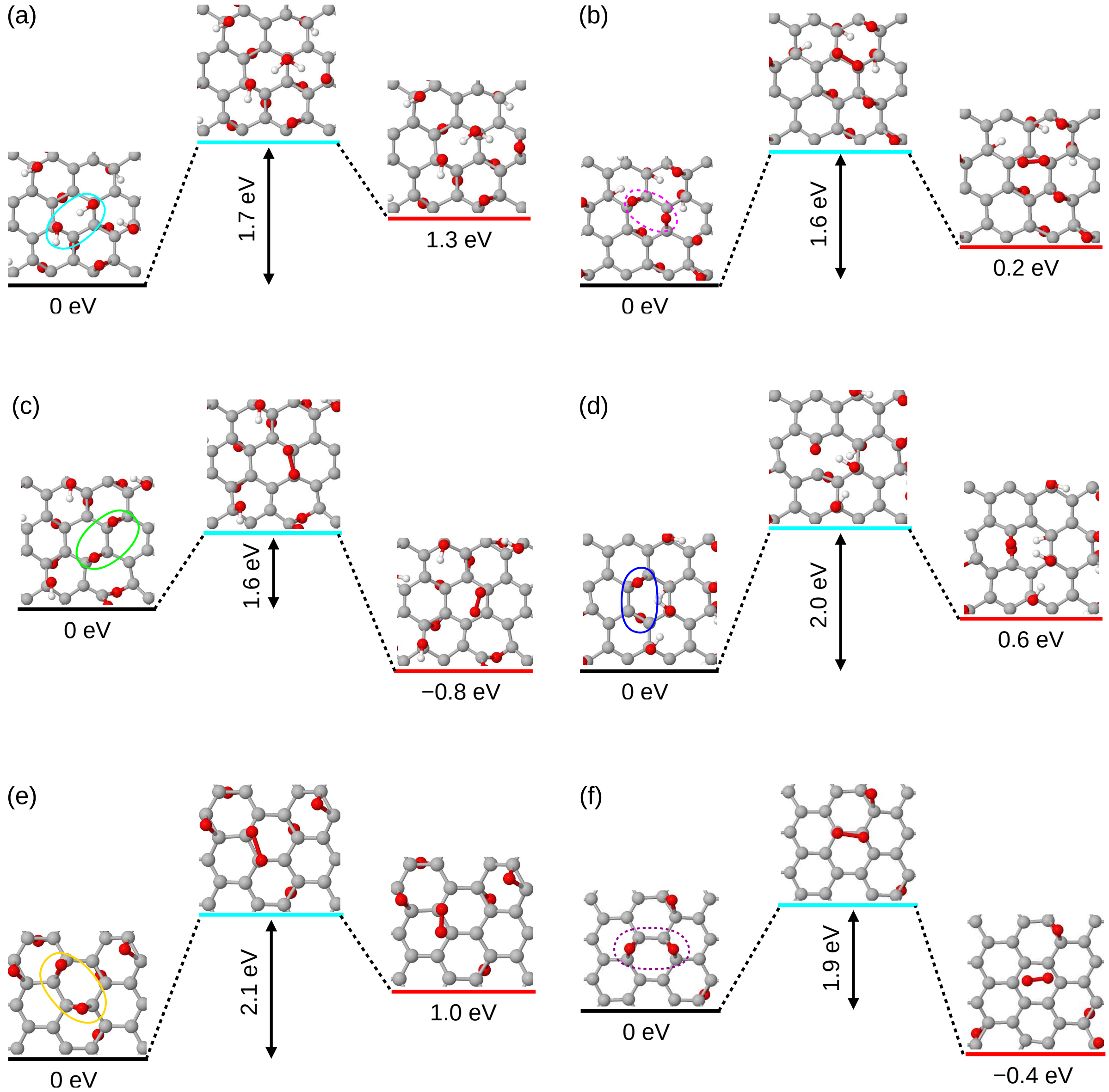

Figure **S13**: **Decomposition reactions of homogeneously functionalized GO**. Energy of reagents (black segments), transition state (cyan segments), and products (red segments) as derived from NEB-DFT calculations of the six reactions shown in Figure S12. (a) Two hydroxyl groups forming a H_2O molecule and an epoxide group. (b) Two epoxide groups forming an O_2 molecule. (c) Same as (b). (d) Two epoxide groups forming a carbonyl pair. (e) and (f), the same as (b). Calculations were based on the use of the two models shown in Figure S12. All the energy values are referred to that one of the initial states.

References

[1] P. Giannozzi, S. Baroni, N. Bonini, M. Calandra, R. Car, C. Cavazzoni, D. Ceresoli, G. L. Chiarotti, M. Cococcioni, I. Dabo, A. Dal Corso, S. de Gironcoli, S. Fabris, G. Fratesi, R. Gebauer, U. Gerstmann, C. Gougoussis, A. Kokalj, M. Lazzeri, L. Martin-Samos, N. Marzari, F. Mauri, R. Mazzarello, S. Paolini, A. Pasquarello, L. Paulatto, C. Sbraccia, S. Scandolo, G. Sclauzero, A. P. Seitsonen, A. Smogunov, P. Umari, and R. M. Wentzcovitch, J. Phys.: Condens. Matter **21**, 395502 (2009).

[2] N. Troullier and J. L. Martins, Phys. Rev. B **43**, 1993 (1991).

[3] J. P. Perdew, K. Burke, and M. Ernzerhof, Phys. Rev. Lett. **77**, 3865 (1996).

[4] G. Henkelman and H. Jonsson, J. Chem. Phys. **133**, 9978 (2000).

[5] C. Zener, Proc. R. Soc. Lond. A **137**, 696 (1932).

[6] W. Orellana, A. J. R. da Silva, and A. Fazzio, Phys. Rev. Lett. **90**, 016103 (2003).
